# Small Order Patterns in Big Time Series: A Practical Guide

**DOI:** 10.3390/e21060613

**Published:** 2019-06-21

**Authors:** Christoph Bandt

**Affiliations:** Institute of Mathematics, University of Greifswald, 17487 Greifswald, Germany; bandt@uni-greifswald.de

**Keywords:** permutation entropy, autocorrelation, time series, order pattern, signal processing

## Abstract

The study of order patterns of three equally-spaced values $x_{t},x_{t+d},x_{t+2d}$ in a time series is a powerful tool. The lag *d* is changed in a wide range so that the differences of the frequencies of order patterns become autocorrelation functions. Similar to a spectrogram in speech analysis, four ordinal autocorrelation functions are used to visualize big data series, as for instance heart and brain activity over many hours. The method applies to real data without preprocessing, and outliers and missing data do not matter. On the theoretical side, we study the properties of order correlation functions and show that the four autocorrelation functions are orthogonal in a certain sense. An analysis of variance of a modified permutation entropy can be performed with four variance components associated with the functions.

## 1. Order Patterns Fit Big Data

### 1.1. The Need for New Methods

A few decades ago, when time series analysis was created, it did suffer from a lack of data. Now, the challenge is an oversupply of data. Modern sensors are inexpensive. They continuously monitor heart and brain signals of patients or athletes, seismic waves, air pollution, radiation, etc. Within one second, hundreds of values can be obtained in one channel. Such time series must be evaluated automatically. To this end, old methods have to be modified, and new ones have to be developed that fulfil the following requirements.

- Basic methods should be simple and transparent.
- Few assumptions should be made on the underlying process.
- Algorithms should be resilient with respect to outliers and artifacts.
- Computations should be very fast.

Classical probabilistic methods have problems with the second property. They often require conditions like a multivariate normal distribution, which are not fulfilled in practice, or formulate their conditions in terms of limits so that they cannot be checked. On the other hand, machine learning procedures are in fashion and very successful for specific purposes, but they do not have the first property. They do not provide clear insight into the observed phenomena.

The analysis of order patterns in a signal fulfills all four requirements. It had been neglected until recently, when the tedious work of counting “larger” and “smaller” in a large series could be delegated to a computer. Today, the use of permutation entropy is well established [1,2,3]. Pattern frequencies have been studied in particular for chaotic dynamical systems (see [2]), correlated noises [4], reversible processes [5,6], and heartbeat RR-intervals [7,8]. In [9], pattern frequencies were studied for Gaussian processes, and the differences of pattern frequencies were introduced as a kind of autocorrelation function. Some applications and the connection with ordinary autocorrelation were discussed in [10]. A new version of permutation entropy was presented in [11].

### 1.2. Contents of the Paper

Here, we continue this work, concentrating on applications to big data series with a multiscale structure. We show that for such applications, order patterns are comparable or even more appropriate than classical methods. In Section 2, we explain the basic calculation of pattern frequencies, including treatment of ties and missing values. Section 3 presents the key concepts of the paper. The properties of order correlation functions are discussed in Section 5, leading to an analysis of variance of permutation entropy. The main purpose of this paper, however, is a proof of concept by application to biomedical signals, to speech and music, and to environmental and weather signals.

### 1.3. A Typical Example

**Figure 1:**
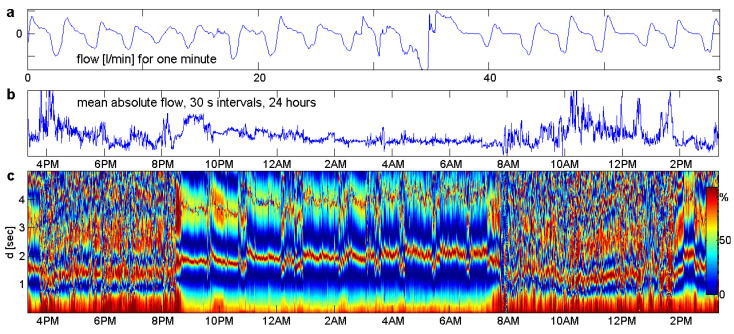
*Our goal is to find structure in big data series.* These are respiration data of a healthy volunteer, measured 50 times per second during 24 h of normal life. (**a**) One minute of clean data. (**b**) Mean flow for intervals of 30 s. (**c**) Order correlation function $\tilde{\tau }\left(d\right)$ for each minute. While Panel b does not provide much information, our method in Panel c shows the differences and tendencies of respiration during the activity phases and sleep stages. Data from a cooperation with Achim Beule.

Figure 1 gives an idea of our intentions. Data came from a cooperation with Achim Beule (University of Münster and University of Greifswald, Department ENT, Head and Neck Surgery). To study respiration in everyday life, two sensors measuring air flow intensity with a sampling frequency of 50 Hz were fitted to the nostrils of a healthy volunteer. This experimental setting was by no means perfect. For example, mouth breathing could not be controlled. As a result, the data contained many artifacts, and even the 3000 values of a “clean minute” shown in Figure 1a looked pretty irregular. Traditional analysis takes averages over 30 s to obtain a better signal, as shown in Figure 1b. Much more information is contained in a function $\tilde{\tau }\left(d\right),$ defined below. Figure 1c shows the collection of these functions for all minutes of the signal, visualized like a spectrogram. We see phases of activity and sleep, various interruptions of sleep, inaccurate measurements around 8:00 and a little nap after 14:00. The frequency of respiration can be read from the lower dark red stripe, which marks half of the wavelength. The upper light stripe marks the full wavelength: 4 s in sleep and 3 s or less in daily life.

## 2. Pattern Frequencies

### 2.1. Basic Idea

We assumed we have a time series $x_{1},x_{2},\ldots ,x_{T}.$ Let d=1 or another small integer, which we call the lag. For any two values $x_{t},x_{t+d}$ that are *d* steps apart, we can decide whether $x_{t}<x_{t+d}$ or $x_{t}>x_{t+d}.$ We can determine the relative frequency $p_{12}\left(d\right)$ of an upward tendency and the complementary frequency $p_{21}\left(d\right)$ of a downward tendency for the lag d. The statistics of these “length two order patterns”, or “ups and downs”, was discussed in [10] and will be embedded here into the statistics of patterns of length three.

**Figure 2:**
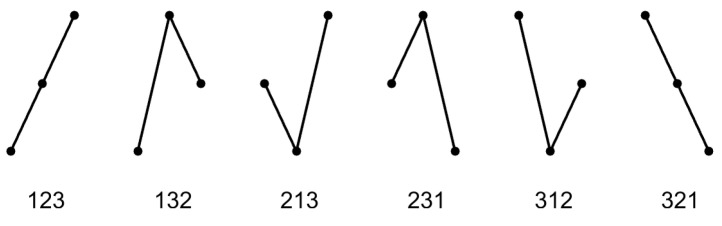
The six order patterns of length three.

To any three equally-spaced values $x_{t},x_{t+d},x_{t+2d}$, we can assign one of the six order patterns in Figure 2. To the pattern π=123, we determine its relative frequency $p_{\pi }\left(d\right)=p_{123}\left(d\right)$ in the time series, and this is done for each pattern π, as explained below. Patterns of length four or longer are more difficult to understand and will not be studied in this paper.

The important point is that we varied the lag or delay *d* — between one and 1000, say. Thus, $p_{\pi }\left(d\right)$ is a function like the autocorrelation function. Both were obtained by a kind of averaging over a certain time period. Our hope was that *such functions will express essential features of the process that generates the data and suppress unimportant individual properties of the observed series.*

### 2.2. Stationarity

The $p_{\pi }\left(d\right)$ are considered as estimates of the probabilities that belong to the underlying process. To justify this viewpoint, we must make a stationarity assumption for the process: the probability of a pattern does not change during time. This is a weak condition, for instance stationary increments in the usual sense will be sufficient [9]. A stronger condition is already needed when we define an average value $\bar{x}$ of the $x_{t}.$

In practice, stationarity is not fulfilled for the complete time series, which in the case of Figure 1 involves more than four million values. For that reason, we always divided the series into short stationary parts. In the biomedical series of Figure 1, each part corresponds to one minute, with 3000 values $x_{t}.$ This is sufficient to estimate the $p_{\pi }\left(d\right)$ in a reliable manner and short enough to expect stationarity. There may be a few minutes where respiration drastically changes, but on the whole, the stationarity assumption is natural and appropriate.

### 2.3. Calculation of Pattern Frequencies

We have the time series $x_{1},x_{2},\ldots ,x_{T}$ with *T* values. While *T* for the whole series will be between $10^{5}$ and $10^{7},$ we now consider only a small stationary part, with typical length *T* between 500 and 20,000. Any three consecutive values $x_{t},x_{t+1},x_{t+2}$ form one of the six order patterns, or permutations, shown in Figure 2. We also considered three values $x_{t},x_{t+d},x_{t+2d}$ with a time lag d>1. The points represent the pattern 231, for instance, if $x_{t+2d}<x_{t}<x_{t+d}.$ If there are ties $x_{s}=x_{t}$ or missing values, the pattern is not defined. The initial time point *t* runs from 1−T−2d. The delay parameter *d* can vary between one and $d_{\max}\leq T/6,$ say, and has the same meaning as in classical autocorrelation. For fixed d, let *n* be the number of time points *t* for which a pattern is defined and $n_{\pi }$ the number of time points where we have pattern π. Then, the pattern frequency is $p_{\pi }\left(d\right)=n_{\pi }/n.$

**Figure 3:**
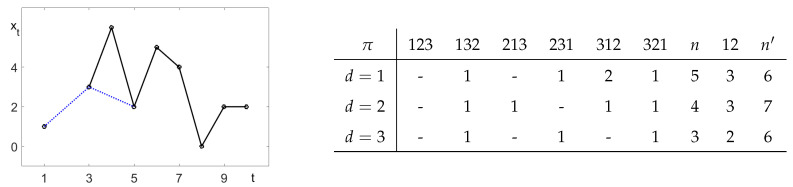
Example time series and pattern frequencies $n_{\pi }.$ The dotted line indicates d=2.

In Figure 3, we have T=10, and the value $x_{2}$ is missing. Thus, t=1 cannot be considered for d=1, but for all d>1. Moreover, there are three equal values $x_{5}=x_{9}=x_{10}$, which exclude t=8 for d=1 and t=5 for d=2. The resulting $n_{\pi }$ are listed in the table. We obtained $p_{312}\left(1\right)=2/5,$ for instance. In Figure 3, we appended the values of $n_{12}$ of the length two order pattern 12. The associated number $n^{\prime }$ of values *t* with $x_{t}\neq x_{t+d}$ was larger than the number *n* for length three.

Since we had only six patterns, the statistics of pattern frequencies was excellent, even for short time series like T=300. For m=6, for instance, we would have 6!=720 patterns, and that would need a very long time series to estimate all those pattern frequencies accurately. Permutation entropy would still work, however, since it is an average over all patterns. The accuracy of estimates is an essential reason to restrict ourselves to patterns of length three.

## 3. Key Concepts and Viewpoints

### 3.1. Permutation Entropy and $\Delta^{2}$

Now, we assume that the pattern frequencies $p_{\pi }\left(d\right)$ are calculated. For each pattern π, they could be drawn as a function of d, like an autocorrelation function. However, it turns out that the pattern frequencies themselves are not so informative. They should be combined in order to form better descriptions of the underlying process. The *permutation entropy* is:

(1) $$H\left(d\right)=-\sum_{\pi }p_{\pi }\left(d\right)\log p_{\pi }\left(d\right)\;,$$

where the sum is taken over the six patterns in Figure 2 or all m! permutations of some lengths m. In other words, we have the probability space of order patterns and take its Shannon entropy. The permutation entropy is a measure of the complexity of the underlying process [12] and has found many applications: distinguishing chaotic and noisy dynamics, classifying sleep stages, detecting epileptic activity from brain signals, etc.

A similar complexity measure introduced in [10,13] is the *distance to white noise:*

(2) $$\Delta^{2}\left(d\right)=\sum_{\pi }{\left(p_{\pi }\left(d\right)-\frac{1}{m!}\right)}^{2}\;.$$

White noise is a series of independent random numbers from a fixed distribution, and it is well known that for this process, all pattern probabilities $p_{\pi }\left(d\right)$ are equal to 1/m! for length m, in particular 1/6 for length three. We just took the quadratic Euclidean distance between the vectors of observed pattern frequencies and the frequencies of white noise. Such distances were studied in [14,15], using relative entropy. There is an even simpler interpretation: the average pattern frequency is always 1/m!, so the *variance of pattern frequencies* equals $\Delta^{2}/m!$. In [11], it was pointed out that $\Delta^{2}$ can be considered as a rescaled Taylor approximation of H, and it has a more convenient scale than H.

### 3.2. Order Correlation Functions

For the six patterns of length m=3, it turns out that four differences of pattern frequencies provide meaningful autocorrelation functions [9,10].

(3) $$\mathrm{up-down}\;\mathrm{balance}\;\beta \left(d\right)=p_{123}\left(d\right)-p_{321}\left(d\right)=p_{12}\left(d\right)-p_{21}\left(d\right)\;,$$

(4) $$\mathrm{persistence}\;\;\tau \left(d\right)=p_{123}\left(d\right)+p_{321}\left(d\right)-\frac{1}{3}\;,\;$$

(5) $$\mathrm{rotational}\;\mathrm{asymmetry}\;\gamma \left(d\right)=p_{213}\left(d\right)+p_{231}\left(d\right)-p_{132}\left(d\right)-p_{312}\left(d\right)\;,\mathrm{and}$$

(6) $$\mathrm{up-down}\;\mathrm{scaling}\;\delta \left(d\right)=p_{132}\left(d\right)+p_{213}\left(d\right)-p_{231}\left(d\right)-p_{312}\left(d\right)\;.$$

The names sound a bit clumsy, and the interpretation of the functions given below is not straightforward. However, it will be shown that these four functions include all information of the six pattern frequencies, that they are orthogonal in a certain sense, and form a variance decomposition of $\Delta^{2}$ given by the Pythagoras-type formula:

(7) $$4\Delta^{2}=3\tau^{2}+2\beta^{2}+\gamma^{2}+\delta^{2}\;.$$

### 3.3. Relative Order Correlation Functions

For the equal pattern probabilities of a white noise process, all terms of this equation are zero. Thus, the definitions were arranged so that white noise was a good null hypothesis for statistical tests. This aspect will not be worked out here. However, the *variance components* of $\Delta^{2}$ were considered as some other ordinal autocorrelation functions and used in some applications, as Figure 1c.

(8) $$\tilde{\tau }=\frac{3\tau^{2}}{4\Delta^{2}}\;,\;\tilde{\beta }=\frac{\beta^{2}}{2\Delta^{2}}\;,\;\tilde{\gamma }=\frac{\gamma^{2}}{4\Delta^{2}}\;,\;\tilde{\delta }=\frac{\delta^{2}}{4\Delta^{2}}\;.$$

A stationary Gaussian process is completely determined by its mean, variance, and autocorrelation function ρ(d), and τ can be directly calculated from ρ [9]. For stationary Gaussian processes, and more generally, for all reversible processes, β,γ, and δ are zero for every d. Thus, our correlation functions can be considered as a tool for non-Gaussian and irreversible processes. The recent papers of Martinez et al. [5] and Zanin et al. [6] defined the degree of irreversibility by comparing all order patterns with their reversed patterns, for different lengths m. Our approach provides three measures of irreversibility, with parameter *d* instead of m. The calculation of β,γ, and δ in our examples will show that processes in practice are mostly irreversible and non-Gaussian.

### 3.4. Two Types of Data

In the literature on permutation entropy and order patterns [1,2,3,4,5,6,7,8,9,12,14], most models and examples act in discrete time: ARMA and GARCH models, discrete dynamical systems, like logistic or Henon maps, and RR-intervals of heartbeat. There is a smallest time unit, and the state of the system changes step by step. As a rule, these models have a strong deterministic component for small d, but a rather short memory. The study of order patterns with length between four and seven makes sense, and is justified by dynamical systems with “forbidden” patterns [2]. On the other hand, a study of large *d* will not reveal a new structure. This also holds for most chaotic systems with continuous time.

**Figure 4:**
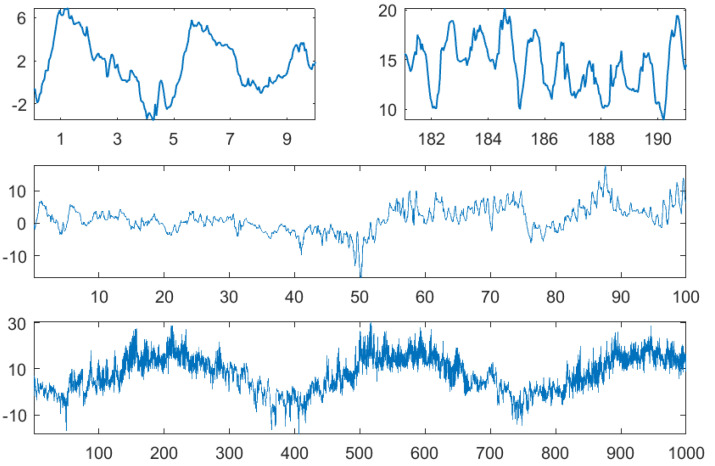
Temperature in ${}^{\circ }$C for the first 10 days in January and July 1978, for 100 and 1000 days.

**Figure 5:**
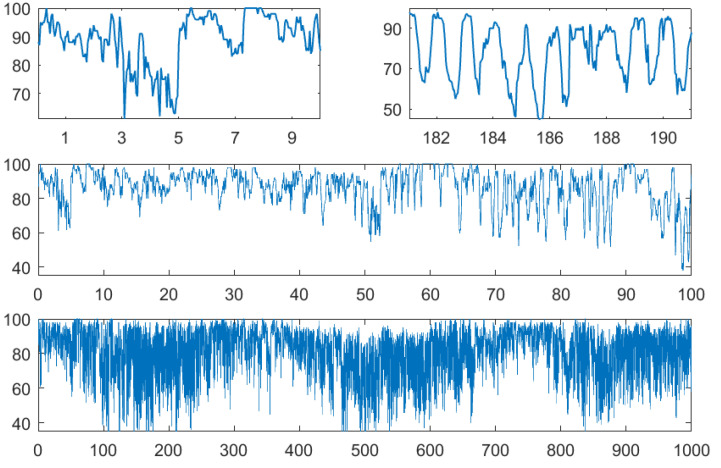
Relative humidity in % for the first 10 days in January and July 1978, for 100 and 1000 days.

The data considered in this paper are different. Continuous measurements in reality generate “big time series” with a multiscale structure, as indicated in Figure 4, Figure 5, 11, and 16 below. Many rhythms with different periods overlap and interact. Below, we mention tides, with a daily, monthly, and yearly scale, and biomedical signals, with scales for heart, respiration, slow biorhythms, day, and night. For these systems, it is crucial to have a scaling parameter *d* with a wide range. The study of patterns with large length *m* and d=1 makes little sense.

Current sensor technology makes it possible to measure with high accuracy almost as fast and as long as we want. Sometimes, a higher sampling rate will reveal new phenomena. For brain signals, 500 Hz can be appropriate, as mentioned in Section 9. In optics, sampling rates today are measured in picoseconds. On the conceptual level, the unit time step is replaced by considering *t* and *d* as continuous parameters assuming arbitrary positive values. We study limits d→0, for instance, even though order correlations are not defined for d=0.

Today’s challenge and bottleneck in time series analysis are the great continuous systems with interacting periodicities and dynamical effects over several scales. Here, we present some simple exploratory tools for their study. Appropriate model classes for such systems are still missing, although there is a well-developed theory of random processes in continuous time. Now let us start with data.

## 4. First Examples: Weather Data

### 4.1. The Data

To demonstrate the use of the above functions, we took hourly measurements of air temperature and relative humidity from the author’s town: Greifswald, North Germany. The German weather service DWD [16] offers such data for hundreds of stations, where results are expected to be similar. Measurements of our data started 1 January 1978. Figure 4 and Figure 5 show temperature and humidity for the first 10, 100, and 1000 days and also for the first 10 days of July 1978. While in summer, there was an obvious day-night rhythm, this need not be so in winter when bright sunshine often comes together with cold temperature. This effect is also visible in the bottom panel with data of almost three years.

**Figure 6:**
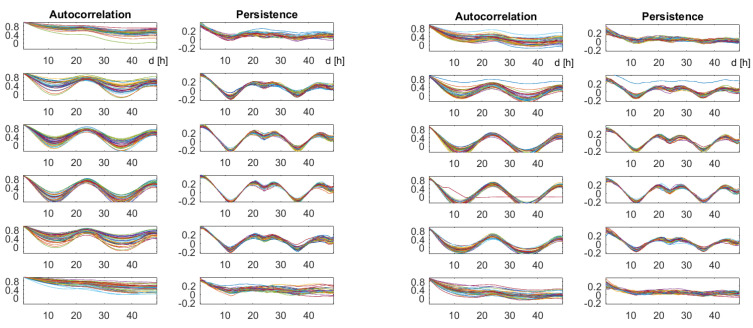
Autocorrelation and persistence for temperature (left part) and relative humidity (right part). The curves correspond to 35 consecutive years. The lag *d* runs from 1–49 h. Each row describes a two-month period, from January/February up to November/December.

We now look for correlation functions that describe the underlying weather process. We calculated them bimonthly, and drew the curves for 35 years for the same season into one plot. When they agreed, they formed a nice description of the properties of the underlying process. Figure 6 compares classical autocorrelation with our persistence function.

### 4.2. Autocorrelation and Persistence

In winter (November to February), a day-night rhythm was not found, and all correlation functions differed greatly over the years. From March until October, the day-night rhythm was well recognized, both by autocorrelation and persistence, and both for temperature and humidity. Classical autocorrelation curves coincided best at the full period maximum at *d* = 24 h, while persistence accurately showed the minimum at the half period of 12 h. At the full period, there was a local minimum between two maximum points, which will be explained in the next section. Both functions succeeded in defining the basic period within each window of two months (1461 h of values). The coincidence over years seemed even better for persistence.

### 4.3. β,γ, and δ

**Figure 7:**
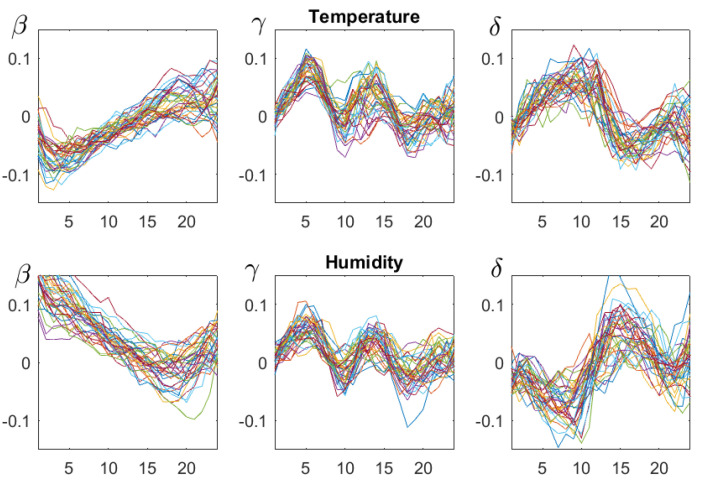
The functions β,γ, and δ, for d=1,…,24 h, for July–August of 35 consecutive years. The upper row corresponds to temperature and the bottom row to relative humidity. Although there is considerable variation over the years, some common structures can be seen.

Is there more structure than the day-night rhythm? Figure 7 shows the functions β,γ, and δ for the summer season (July–August). In summer, the air warms up fast and cools down slowly, which explains why β was negative for small d. Humidity increases slowly and decreases fast, so β was positive for small d. In view of Equation (11) below, the δ functions also showed the opposite behavior. This was found for all seasons. The structure of γ, which was similar for temperature and humidity, was found only in spring and summer (April–August), and we have no interpretation for it. It seems that these functions can be used to characterize sites.

## 5. Properties of Correlation Functions

### 5.1. Two Pattern Identities

For fixed d, the basic equation for $p_{\pi }=p_{\pi }\left(d\right)$ is:

(9) $$\sum_{\pi }p_{\pi }=1\;\mathrm{for}\;\mathrm{fixed}\;d\;\mathrm{and}m\;.$$

The following equation is accurate for probabilities in a stationary process [9].

(10) $$p_{123}+p_{132}+p_{231}=p_{12}=p_{123}+p_{213}+p_{312}\;.$$

To show equality on the left, divide the probability $P(x_{t}<x_{t+d})$ into three terms, depending on whether $x_{t+2d}$ is above, between, or below $x_{t}$ and $x_{t+d}.$ For the equality on the right, we considered $P(x_{t+d}<x_{t+2d})$ and summed up three cases for $x_{t}.$

### 5.2. Marginal Errors

If we calculate the frequencies for a time series with d=1, then $p_{12}$ is an average over t=1,…,T−1. To determine $p_{12}$ from the three patterns on the left, we averaged over t=1,…,T−2. When we took the three patterns on the right, we averaged over t=2,…,T−1. There was a marginal difference. Moreover, the exclusion of missing and equal values can be different for patterns of different lengths, as demonstrated above. Such marginal effects were the reason for the difference in the two expressions for β in (3). Actually, (3) is a corollary of (10) and $p_{321}+p_{312}+p_{213}=p_{21};$ see [9].

As an example, we determined for Figure 3 the frequencies of the patterns 12 and 21, that is $x_{t}<x_{t+d}$ and $x_{t}>x_{t+d}.$ For d=1, we had $n_{12}=n_{21}=3,$ and for d=2, we obtained $n_{12}=3,n_{21}=4.$ When we determined $\beta \left(d\right)=p_{12}\left(d\right)-p_{21}\left(d\right),$ we obtained β(1)=0,β(2)=−1/7. If we used the definition $\beta \left(d\right)=p_{123}\left(d\right)-p_{321}\left(d\right)$ in (3), we obtained β(1)=−1/5,β(2)=−1/4.

For T≥300 and d=1 or 2, such marginal effects are really harmless. For large d, however, they can become larger since the margins of (10) will involve *d* time points on the right and left end of the time series. This can be a problem when we have a downward trend at the beginning of the series and an upward trend at the end. For EEG brain data, we helped ourselves by defining the parts of the large series not by equal length, but by zero crossings of the time series from the positive to the negative side [11]. Other data may require other solutions for improved estimates of pattern frequencies.

### 5.3. Classical Autocorrelation

The classical autocorrelation function ρ (see [17,18]) measures the degree of coincidence of the time series with a copy shifted by *d* time steps. Thus, autocorrelation is large for small d. It will decrease, and the rate of decrease reflects the memory of the underlying process. If we have a periodic dynamics, autocorrelation will be large at the period, and small, mostly negative, at the half period. Moreover, the sign of ρ can indicate an increasing or decreasing trend. The last point is not quite correct, because a trend will exclude stationarity, and then, strictly speaking, we cannot estimate autocorrelation.

### 5.4. Interpretation of β,γ,δ

The direction of a trend is always indicated by β, and this can be consistent with the weak stationarity assumptions to estimate order pattern frequencies, for instance in the case of stationary increments with non-zero mean. The main purpose of β, as well as of γ and δ is the description of certain asymmetries in the process. The up-down balance β is positive for small *d* if the process spends more time with increase than with decrease. When the process is stationary, this means that values will increase more slowly and decrease faster. The functions γ and δ are more difficult to interpret, but we can at least say that δ is tightly connected to β since:

(11) δ(d)=β(2d)−β(d).

This follows from $\beta \left(2d\right)=p_{123}\left(d\right)+p_{132}\left(d\right)+p_{213}\left(d\right)-p_{321}\left(d\right)-p_{312}\left(d\right)-p_{231}\left(d\right)$ and from the definition of β. As in Section 2, the equation holds for pattern probabilities of a process and may have a marginal error for frequencies of a concrete time series. This equation justifies the name up-down scaling for δ.

### 5.5. Persistence and Turning Rate

Persistence is the most common and most important of our functions. It says how often a relation $x_{t}<x_{t+d}$ or $x_{t}>x_{t+d}$ will persist in the next comparison of $x_{t+d}$ with $x_{t+2d}$. Up to the sign, it can replace autocorrelation. As shown in [10] (Section 4), it can be interpreted similarly, and is less susceptible to noise and nonlinear distortion. For small d, persistence measures the degree of smoothness of the time series. When we have a smooth function, then τ(d) tends to the maximal value $\frac{2}{3}$ for d→0. We can also consider the complementary quantity:

(12) $$\mathrm{turning}\;\mathrm{rate}\;\;TR=p_{132}\left(d\right)+p_{213}\left(d\right)+p_{231}\left(d\right)+p_{312}\left(d\right)=\frac{2}{3}-\tau \left(d\right)\;,\;$$

which counts the frequencies of turning points (local maxima or minima) in the series. Obviously, TR is a measure of roughness, or variation [19].

In this paper, however, the functions were chosen so that they were all zero for white noise; and for the slightly more general case of an exchangeable process where all patterns of Figure 2 had the same probability $\frac{1}{6}.$ In other words, white noise was the origin point of our coordinate system. This viewpoint was especially useful when we dealt with noisy signals, for instance EEG brain data. All our functions were the differences of pattern frequencies. For τ, we have:

(13) $$\tau \left(d\right)=\frac{1}{3}\left(2p_{123}\left(d\right)+2p_{321}\left(d\right)-p_{132}\left(d\right)-p_{213}\left(d\right)-p_{231}\left(d\right)-p_{312}\left(d\right)\right)\;.$$

Both (12) and (13) can be easily checked with (9). In the following, we systematically summarize the properties of autocorrelation functions. For the sake of completeness, the next tables include Spearman’s rank correlation, but not Kendall’s τ [20]. We think that for big time series, persistence better reflects Kendall’s idea to measure correlation by order comparison. This is why we used the letter τ.

Table 1 shows the range of values for all correlation functions, adding for which series minimal and maximal values were attained. An alternating series changes up and down at each step, like $x_{t}={\left(-1\right)}^{t}+0.1\cdot \left(\mathrm{uniform}\;\mathrm{white}\;\mathrm{noise}\right)$, where the noise is added to avoid equal values. This works for d=1, not for continuous d. For γ and δ, we just gave an example series, which may give a feeling for what these functions measure.

### 5.6. Symmetries of Order Functions

A function f(d) is even if f(−d)=f(d) and odd if f(−d) = −f(d). In the case of order correlation functions, even functions are those that assume the same values for a time series $x_{1},x_{2},\ldots ,x_{T}$ and its time-reversed series $x_{T},x_{T-1},\ldots ,x_{1}.$ For odd functions, time reversal changes the sign of the correlation function. We can also ask for invariance under the change of sign of the series, which gives $-x_{1},-x_{2},\ldots ,-x_{T},$ or for the combined change of sign and time reversal, $-x_{T},-x_{T-1},\ldots ,-x_{1}.$ The latter can be interpreted as a $180^{\circ }$ rotation of the graph of the series.

All autocorrelation behaved well under these symmetries. They either remained invariant, in which case we wrote a + in Table 2, or they changed sign, in which case we wrote −. Persistence is an even function like ρ, but our functions β,γ, and δ were odd. This caused a discontinuity at d=0 unless the value was zero there. Actually, none of our correlation functions was defined for d=0. We simplified matters by restricting ourselves to strictly positive d. However, the problem will return when we consider periodic series.

### 5.7. Periodicities

A time series $x_{1},\ldots ,x_{T}$ is periodic with period *L* if $x_{t+L}=x_{t}$ for t=1,…,T−L. Theoretically, none of our correlation functions was then defined for d=L because there were only equal values to compare. In reality, however, we had only approximate periodicity and could always determine values for d=L. Nevertheless, some phenomena occurred at d=L,2L,3L,… and at half periods $d=\frac{L}{2},\frac{3L}{2},\ldots$ For a periodic time series, all autocorrelation functions were periodic with the same period; in practice, only approximately, since only part of the generating mechanism worked periodically. Therefore, it sufficed to consider d=L and $d=\frac{L}{2}$ in Table 3.

For an even function f(d) with period *L*, we had f(d)=f(L−d), and hence, $f\left(\frac{L}{2}+k\right)=f\left(\frac{L}{2}-k\right).$ That is, an even correlation function is mirror-symmetric with respect to the vertical line d=L, as well as $d=\frac{L}{2}.$ This is the case for ρ and τ. For an odd function g(d) with period *L*, we had g(L−d)=−g(d), and hence, $g\left(\frac{L}{2}+k\right)=-g\left(\frac{L}{2}-k\right).$ In particular, $g(\frac{L}{2})=0.$ The graph of *g* then had a symmetry center at (L,0) and at $(\frac{L}{2},0).$ Both symmetry centers will be seen as zero crossings in the graphs of β,γ, and δ even though theoretically, we may have a discontinuity at L. For noisy series, the study of symmetries of correlation functions can be quite helpful to recognize periodicities.

Let us now explain the behavior of persistence, which we have already seen in Figure 6. For $d=\frac{L}{2},$ we always had $x_{t}\approx x_{t+2d}$ in a series with approximate period L. Thus, if $x_{t+d}$ was only a bit larger or smaller, we could not have the pattern 123 or 321. This means that τ had a clear minimum at $\frac{L}{2}.$ If the noise level went to zero, the value would approach the absolute minimum $-\frac{1}{3}.$ This is the best way to determine a period with τ.

At d=L, the behavior was more complicated and different from the maximum of ρ. Assume that much of the time series consists of monotone pieces and $x_{t}$ is on an increasing branch. If we had the exact period *L* and took d=L−ε for a small ε>0, then $x_{t}>x_{t+d}>x_{t+2d}$, since the shifted values were further downwards on the repeating increasing branch. However, for d=L+ε with small positive ε, we had $x_{t}<x_{t+d}<x_{t+2d}$, since now the shifted values came further upwards on the repeating branch. Only for d=L, we theoretically had $x_{t}=x_{t+d}=x_{t+2d}$ and can say nothing. In practice, there will be some noise that disturbs the monotone branches and changes our conclusion for small ε. Anyway, left and right of d=L, the patterns 123 and 321 dominated, and we had two maxima of persistence there. However, for d=L itself, the patterns 123 and 321 did not dominate anymore, and we obtained a local minimum of τ. This is what we call a bumped maximum. The height and width of the bump decreased when the noise level went to zero. See Figure 6.

One can ask why among our four correlation functions, only τ was even and the other three were odd. Actually, we had six order patterns, so we could have three even and three odd functions. However, the patterns fulfilled the sum condition (9) and another condition, which directly follows from (10):

(14) $$p_{132}+p_{231}-p_{213}-p_{312}=0\;.$$

Both of these conditions are expressed by even functions of d, since the frequencies of a permutation and its reversal, like 213 and 312, have the same sign in the equation. Thus, we were left with four degrees of freedom for our pattern frequencies, and it is natural to obtain three odd correlation functions.

### 5.8. The Decomposition Theorem

Now, we show that τ,β,γ, and δ are an optimal choice of correlation functions, which explains in some way all the difference between our observed time series and white noise, a series of independent random numbers. The information given by these functions is largely independent, with the exception of dependence between β and δ, which cannot be avoided. This will be made precise in a subsequent mathematical paper. Here, we argue that certain vectors corresponding to the functions are orthogonal, which can be the basis for ANOVA (analysis of variance) techniques.

**Theorem** **1** (Pythagoras theorem for order patterns of length three)**.** *For a process with stationary increments and an arbitrary lag d, the quadratic distance $\Delta^{2}$ of pattern probabilities to white noise uniform pattern frequencies $\frac{1}{6}$ defined in (*2*) has the following representation:*

(15) $$4\Delta^{2}=3\tau^{2}+2\beta^{2}+\gamma^{2}+\delta^{2}\;.$$

**Proof.**  For arbitrary numbers $q_{1},q_{2},\ldots ,q_{6}$, we have the following identity.

$$\begin{array}{ccc} 4\sum_{k=1}^{6}q_{k}^{2}= & 2{\left(q_{1}+q_{6}\right)}^{2}+2{\left(q_{1}-q_{6}\right)}^{2} & +{\left(q_{2}+q_{3}+q_{4}+q_{5}\right)}^{2}+{\left(q_{2}-q_{3}-q_{4}+q_{5}\right)}^{2}\\ & & +{\left(q_{2}+q_{3}-q_{4}-q_{5}\right)}^{2}+{\left(q_{2}-q_{3}+q_{4}-q_{5}\right)}^{2} \end{array}$$

If $\sum q_{k}=0,$ the third square on the right is the same as the first. Now, let $q_{1}\;=\;p_{123}\;-\;\frac{1}{6},q_{2}\;=\;p_{132}-\frac{1}{6},q_{3}=p_{213}-\frac{1}{6},q_{4}=p_{231}-\frac{1}{6},q_{5}=p_{312}-\frac{1}{6},$ and $q_{6}=p_{321}-\frac{1}{6}.$ Then, $\sum q_{k}=0$ by (9), and the last term on the right is zero by (14). According to the definitions of $\Delta^{2},\tau ,\beta ,\gamma ,$ and δ, the identity turns into Equation (15) of the theorem. This completes the theoretical part of the paper. □

## 6. Case Study: Speech and Music

One potential field of application of our correlation functions is speech and music: speech recognition, speaker identification, emotional content of speech sounds, etc. This field is dominated by spectral techniques and by machine learning, and additional information on speech processes is certainly welcome. We are not going into detail, since we know this is hard work.Here, we just analyze the first 12 s of the song “Hey Jude” by The Beatles, to indicate what is possible.

### Sliding Windows

**Figure 8:**
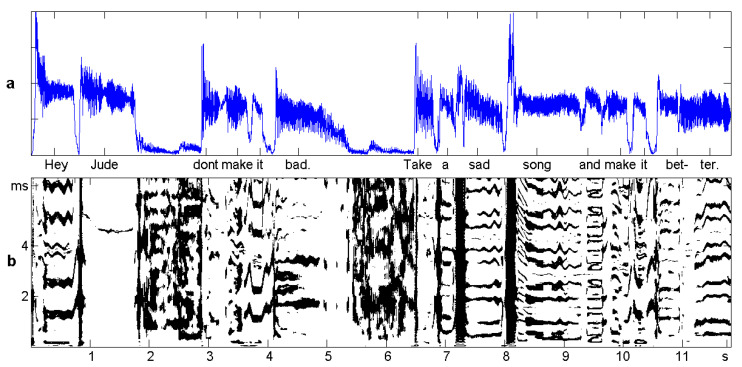
Twelve seconds of the song “Hey Jude” by The Beatles. (**a**) The signal: mean of the absolute amplitude over non-overlapping windows of 50 ms. (**b**) The noisy places (x,d) for which $T\Delta^{2}<15,$ drawn in black. The vertical axis represents the lag d=1,…,30, considered as the wavelength, which ranges from 0–7 ms. Each column of the matrix corresponds to one window x.

The intensity of the signal is shown in Figure 8. Since music is sampled at 44 kHz, there are half a million amplitude values. For a rough analysis, we divided the large time series into 240 non-overlapping pieces, called windows, of length 50 ms. Thus, there were 240 time series of length T=2200 for which we could determine the mean absolute amplitude and correlation functions. The delay *d* ran from 1=1/44 ms to d=300, which is 7 ms. The functions will not be drawn as curves, but as color-coded vertical sections of an image, which describes the whole dataset. The windows, or columns of the matrix, are numbered x=1,…,240 and the rows d=1,…,300, written as 0.7 ms.

As a first experiment, we chose the points (x,d) for which $T\Delta^{2}<15.$ They are colored black in Figure 8b. At these places, we could perhaps still confuse the signal with white noise; according to the simulations in [11], the *p*-value was larger than $10^{-15}.$ These places occupied 26% of the matrix, notably the “k” of “take” and “s” of “sad” and “song” with almost any d. For all the other (x,d), Theorem 1 says that some of our correlation functions are significant.

**Figure 9:**
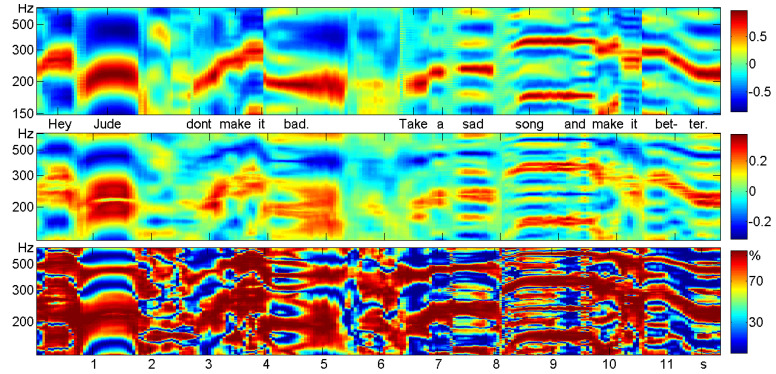
Correlogram (upper panel) and persistence (middle panel) of 12 s of “Hey Jude”. The scale of *d* was reverted and written as frequencies so that the melody could be read like musical notes. The bottom panel shows the percentage $\tilde{\tau }$ of $\Delta^{2}$, which is due to persistence.

Sliding window analysis of speech and music and visualization in a matrix is well known from the spectrogram, where columns correspond to the Fourier spectrum of the windows. Since here, we were satisfied with the melody (the so-called pitch frequency of the singer), we took the correlogram instead and compared with persistence in Figure 9. For convenience, the *d*-scale was transformed into frequencies. So 500 Hertz corresponds to d=2 ms, and 200 Hz means d=5 ms. The maxima of ρ and the bumped maxima of τ were very clear for all voiced sounds. They described the melody, and they did coincide.

**Figure 10:**
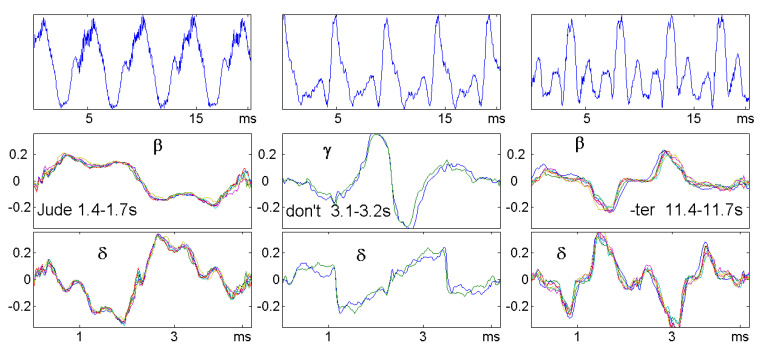
Detail from Figure 9. The vowels of “Jude”, “don’t”, and the second syllable in “better” provide stationary parts of the time series lasting for 0.3, 0.1, and 0.3 s. Only 20 ms of each signal are shown in the top panel. Order correlation functions were calculated for six, resp. two, disjoint windows of a length of 50 ms and drawn for one pitch period, which equaled 4.5 ms for all three sounds.

The bottom panel of Figure 9 shows $\tilde{\tau }=\frac{3\tau^{2}}{4\Delta^{2}},$ the percentage of $\delta^{2}$ that is due to persistence. Clearly, persistence was the dominating function, with 80% of $\Delta^{2}$ at most places. However, there were also many places with 30% and less where the functions β,γ,δ formed the larger part of $\Delta^{2}.$ Figure 10 shows some phonemes that may be characterized this way.

## 7. Case Study: Tides

### 7.1. The Data

**Figure 11:**
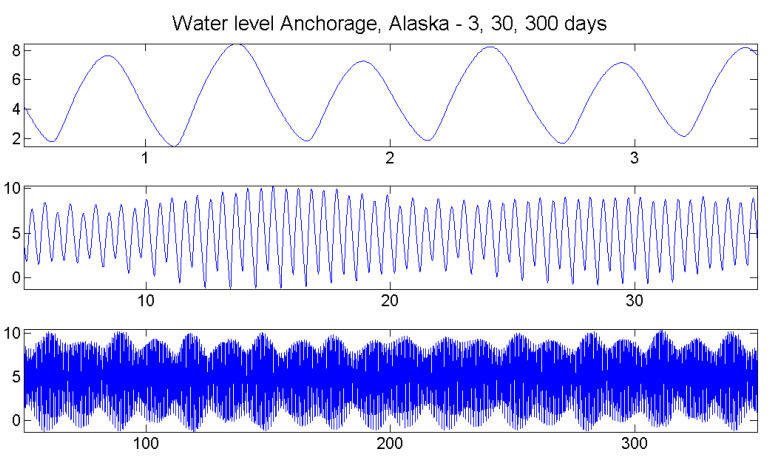
Tides form an almost deterministic process with periodicities on the scale of days, months, and years. Data from [21].

Tides of the oceans are a well-studied phenomenon, and tidal physics is a science that has developed over centuries. They form a good testbed for our correlation functions for two reasons. On the one hand, there are excellent data series of water levels at many stations, provided for the U.S. by the National Water Level Observation Network [21] over many years with 6-min intervals and few missing values. On the other hand, tides can be considered as an almost deterministic process driven by the interaction of the Moon, Sun, and Earth, with geographical site and coastal topography of the station as parameters. As Figure 11 shows, there are daily, monthly, and yearly scales of periodicities: tides at a place come two times a day, spring tides two times a month, etc.

**Figure 12:**
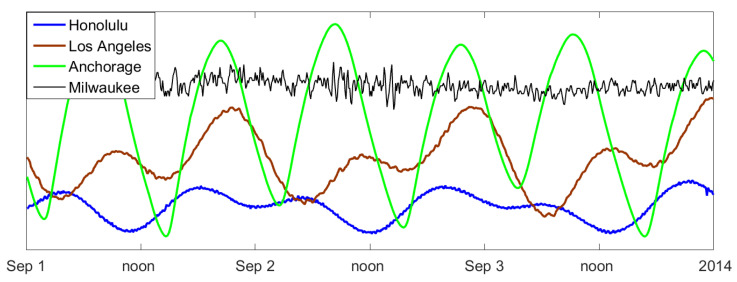
Water levels for three days in 2014, measured every six minutes at different stations in the U.S. Data taken from [21]; shifted and scaled for better visibility.

Since Anchorage, Alaska, is situated at the end of a narrow bay, the tidal range (difference between high tide and low tide) is 8 m. In Los Angeles, California, it is less than 2 m, and in Honolulu, Hawaii, it is only half a meter. Order patterns cannot distinguish the size of waves; we are only interested in their structure!Moreover, it makes no sense to compare zero levels of stations, so they were shifted for better visibility. For the sake of completeness, Figure 12 includes Milwaukee on Lake Michigan, where there are no tides and the differences are only 10 cm, caused by changes of wind and barometric pressure. This series shows what fluctuations arise when data represent random weather change.

### 7.2. Order Correlation

**Figure 13:**
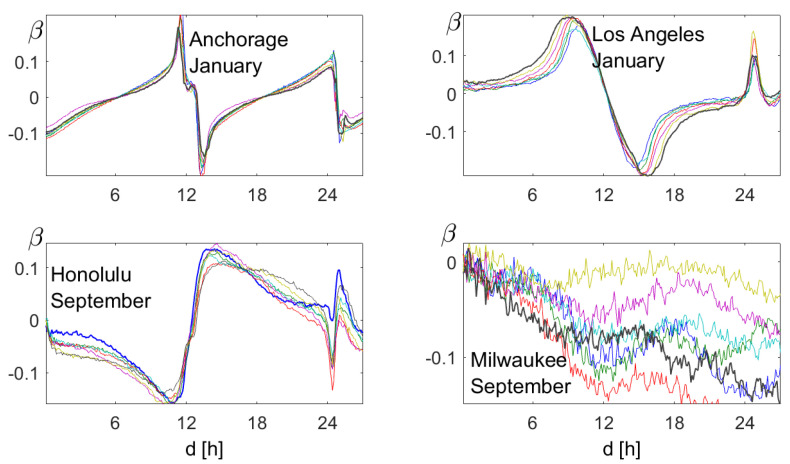
Correlation functions β, for one month in seven consecutive years, at the four places of Figure 12. For a given month, each ocean station has its specific β-profile.

The functions β given in Figure 13 showed that each of the ocean stations had its own profile, which did not change much in seven consecutive years. It did depend a bit on the season, however, so it was taken for one month in each year. Since we had 240 values per day, we could afford even shorter periods.

Experience shows that water comes fast and goes slowly, so β should be negative for small d. This is clearly true for Milwaukee, where no periodicity was found, and for Honolulu. For Anchorage and Los Angeles, it was the other way round. For the three ocean stations, β had a zero-crossing for d≈12.5 and a “discontinuity” at d=25. As explained in Section 5, this corresponds to the basic daily periodicity of 24.8 h (the difference from 24 is caused by the Moon).

**Figure 14:**
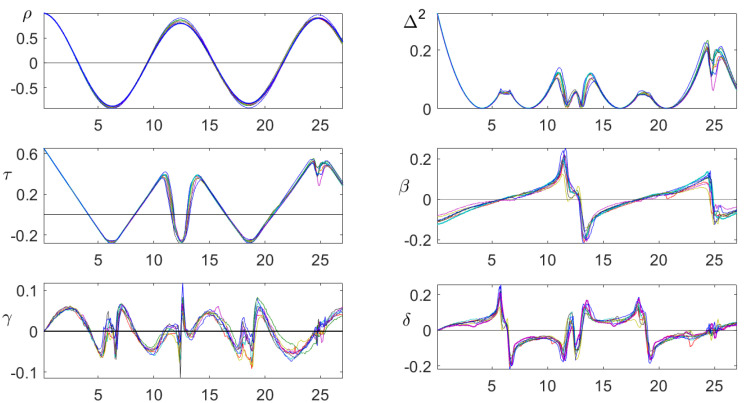
Classical autocorrelation and order correlation functions for the station at Anchorage in January. Persistence reflects the diurnal rhythm. The asymmetry functions β,γ,δ all show a very specific structure, which remains stable through seven consecutive years. In contrast, ρ does not seem to contain much structural information.

Figure 14 shows all order correlations for Anchorage for the month January. Both the autocorrelation and persistence expressed the fact that there were two periodicities with periods of 12.5 and 25 h. For ρ, the maximum at 25 was larger than the first maximum at 12.5, while the minimum at 19 was not smaller than the first minimum at six. For persistence, the maximum at 25 had a small bump, while the maximum at 12.5 had a very large bump, since it was also a minimum corresponding to 25. The functions β,γ,δ all showed a sharp structure that was preserved through consecutive years. This is no proof that they contain more information than ρ, but it is an argument for further study of these functions.

### 7.3. Relative Order Correlation

**Figure 15:**
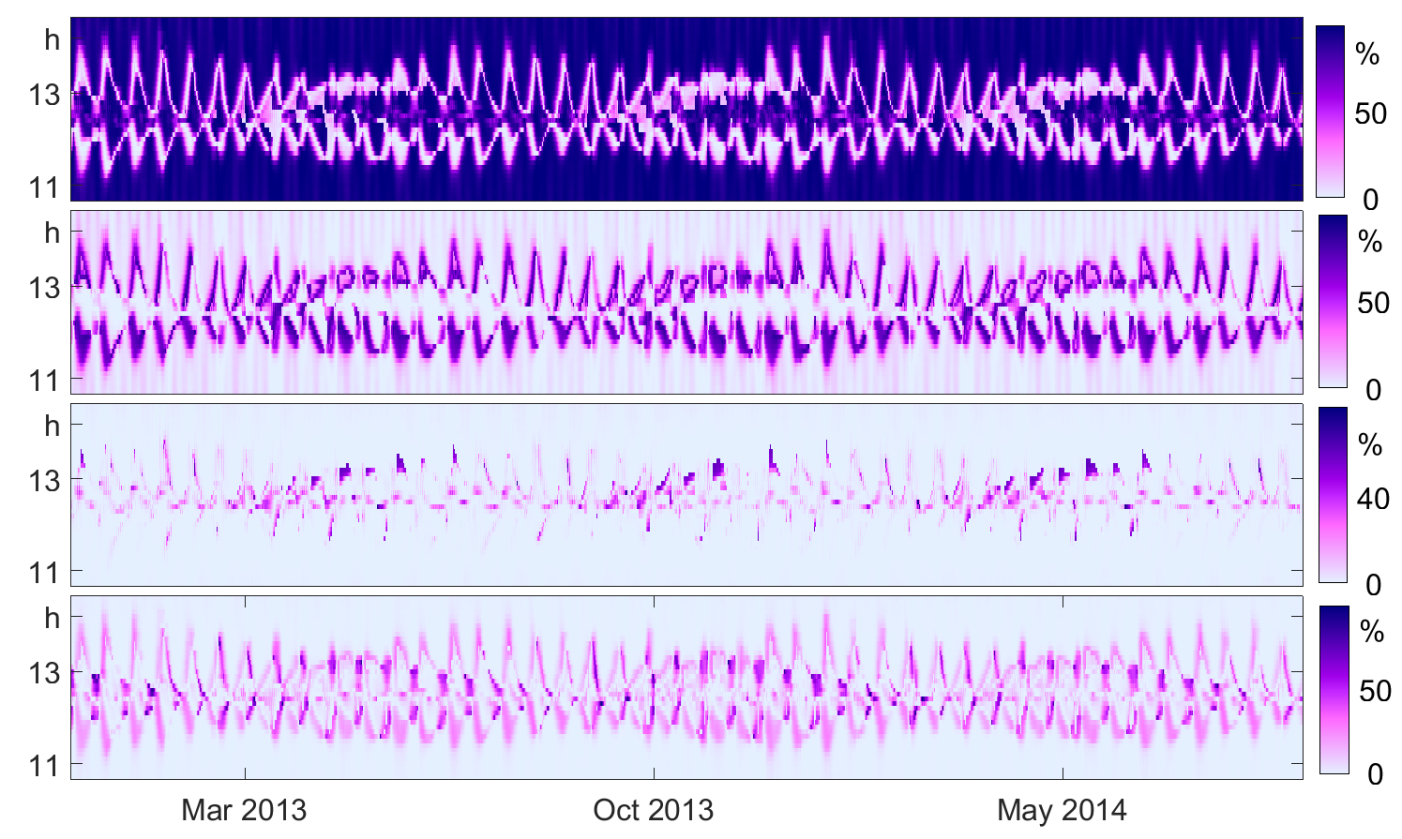
The division of $\Delta^{2}$ into the components described in Theorem 1, illustrated for the tides at Anchorage in the years 2013 and 2014 in sliding window analysis. From top to bottom, the four panels correspond to the function $\tilde{\tau }$, which takes the largest part of $\Delta^{2},$ to $\tilde{\beta },\tilde{\gamma }$, and $\tilde{\delta }.$

Let us check Theorem 1 with the tides data of Anchorage in Figure 15. Windows of length 1242 (five days and 4.2 h, just five main periods) were used for the years 2013 and 2014. For good resolution, the step size was half a day, so successive windows overlapped by 9/10 of their length. The lag *d* was restricted to the time between 10.5 and 14.5 h, around the first important period of 12.5 h where $\Delta^{2}$ was large. Roughly speaking, the picture shows how the position and width of the main bump of τ in Figure 14 changed with time. There were small waves that described the bimonthly period and long waves describing biyearly periodicity.

It can be seen how the different components fit together, with $\tilde{\tau }$ as the dominant component. The average value of $\tilde{\tau }$ was 76%, followed by 15% for $\tilde{\beta },$ 6% for $\tilde{\delta },$ and 2% for $\tilde{\gamma }.$ Since Theorem 1 holds for the probabilities of the process and here we have frequencies from data, there was a marginal error, as explained in Section 3. Since *d* was between 105 and 145, this error could be rather large. Here, the average error was 0.5%. There were very few places (x,d) where the error was more than 1%, the maximum being 2%.

This example shows that the order correlation functions $\tilde{\tau },\tilde{\beta },\tilde{\gamma }$, and $\tilde{\delta }$ can also detect structure in data, even though they are squares and lack the information of the sign of the original functions. In the present case, as well as in Figure 1c, these relative quantities were more informative than the original ones.

## 8. Case Study: Particulates

### 8.1. The Data

**Figure 16:**
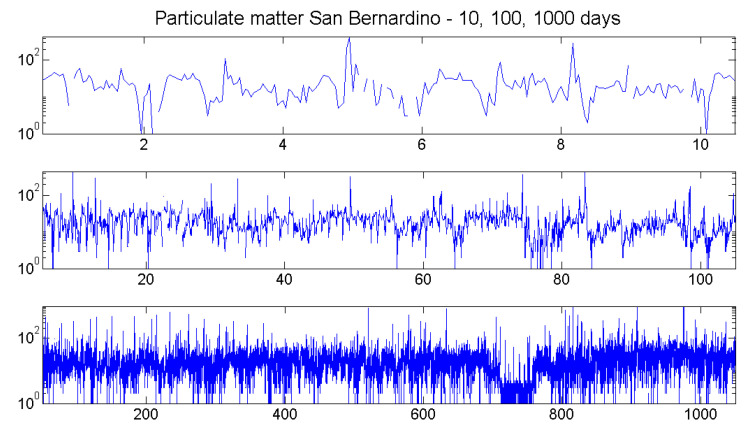
Particulate measurements are notoriously noisy. They show a weak daily and yearly rhythm, which can hardly be detected from the data. The PM10 measurements for Station 3215 Trona-Athol in San Bernardino, California, are from the public database [22].

In the previous section, we had an almost deterministic process with excellent data. Now, we consider the opposite situation: measurements of particulates PM10 (aerodynamic diameter smaller than 10μm). Such data, measured by a kind of vacuum cleaner with a light sensor for the dust, are notoriously noisy. Dust will not come uniformly; it will rather form clusters of various sizes. Although measurements can be taken several times in a second, they are averaged automatically at least over several minutes. In Figure 16, we consider hourly values from the public database [22] for Station 3215 at Trona-Athol in San Bernardino, California. They were transformed to logarithmic scale to reduce the influence of large values. Such a transform does not change order patterns.

**Figure 17:**
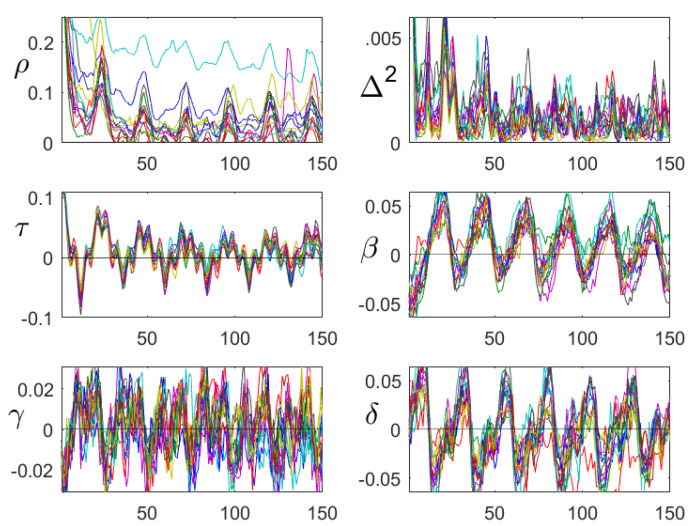
Correlation functions for hourly particulate measurements in San Bernardino [22] in 2000–2011. The 12 curves correspond to years and show the consistency of the correlation structure over the years. The order correlation functions keep the structure from Days 1–6 better than autocorrelation.

There were 13% missing values, some of which can be detected in the upper panel of Figure 16. They affected the estimation of autocorrelation much more than the estimation of order correlation functions. This can be seen in Figure 17, where all functions were determined for 12 successive years. The variation was of course a bit larger than in the corresponding Figure 14 for the tides. The values of the order correlations were about three-times smaller than those for the tides. The important point, however, is the consistent structure of the order correlation functions. It was much better than classical autocorrelation or $\Delta^{2}$ in the upper row. Persistence showed a loss of intensity from one. up to six days, though not as strong as autocorrelation. However, the structure of β,δ, and even γ remained almost unchanged from Days 1–6.

### 8.2. Sliding Windows Analysis

**Figure 18:**
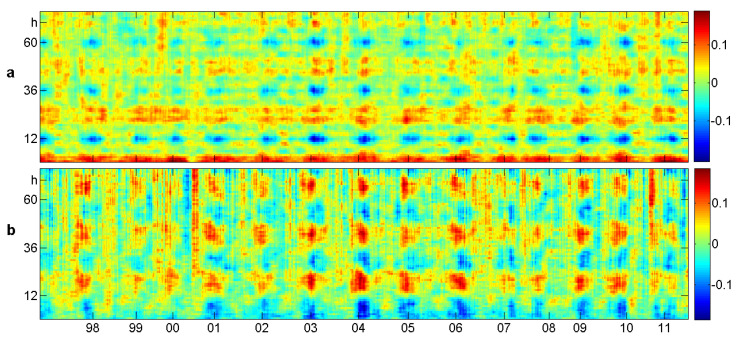
Sliding window analysis of (**a**) persistence τ(d) and (**b**) up-down balance β(d) for hourly particulate measurements in San Bernardino 1997–2011 [22]. The lag *d* runs from 1–72, that is three days, on the vertical axis. Overlapping windows of 50 days were used. Daily rhythm is present mainly in summer, in both τ and β.

We took windows of length 1200, that is 50 days. Smaller windows do not provide sufficiently accurate estimates. Probably, the situation would be better if the data were measured every 6 min, like the tides above, even though there were larger variations. Figure 18 shows that the daily rhythm appears mainly in summer, not in winter, similar to temperature in Section 4. Thus, the curves of Figure 17 were obtained mainly from the summer measurements. The strength of daily rhythm and the “length of summer season” varied over the years.

## 9. Brain and Heart Signals

### 9.1. The Data

**Figure 19:**
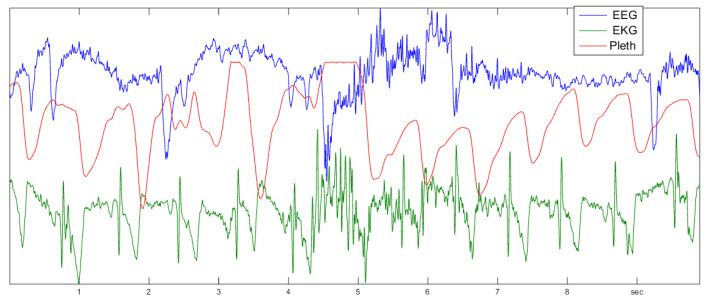
Biomedical signals: 8 s of an electroencephalogram, an electrocardiogram, and a plethysmogram. Order correlation functions seem to apply to all of them.

The examples in Section 4, Section 7, and Section 8 were presented as a proof of concept, with the idea to encourage readers to work out such applications more carefully. For biomedical data, however, the author has done a detailed study [11,13,19] of data published by Terzano et al. [23] at physionet [24], which seems very promising. Three types of data were considered, as indicated in Figure 19: the noisy EEG brain data (electroencephalogram), the well-known heart ECG (electrocardiogram), and the rather smooth plethysmogram, which measures the blood flow at the fingertip.

### 9.2. Sleep Stages

**Figure 20:**
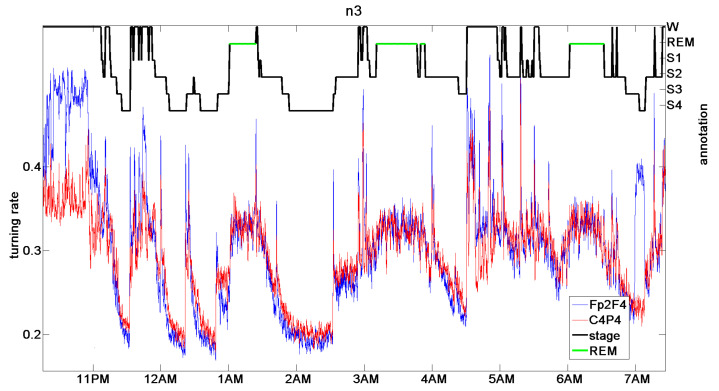
Coincidence of sleep stage annotation by a medical expert and turning rate for d=8ms of two EEG channels [19]. Original data from Terzano et al. [23].

In [11], it was found that the function $\Delta^{2}$ can distinguish sleep stages from the EEG data without any further processing. In [19], it was noted that it is actually the persistence, or turning rate, the main component of $\Delta^{2},$ that can be taken as the measure of sleep depth. For all healthy control persons and a number of patients with different sleep problems, the results were as impressive as Figure 20, where manual annotation of sleep stages (wake, S1 to S4, REM sleep) by a medical expert and turning rate were almost parallel. The definition of sleep depth is a complicated and controversial issue among physicians, and a pure mathematical definition, even if it is not perfect, could be helpful.

So far, the author has not been able reproduce this result with more recent data. Actually, there are huge databases of sleep data in different countries. However, these data are not of the same quality as those of [23] for two reasons. On the one hand, since there are so many data, they are sampled not at 500 Hz, but only at 200 Hz or less. On the other hand, EEG data are contaminated by the field of the electrical power net with a 50–60-Hz frequency. It is quite an effort to avoid this influence, especially in a hospital environment. Since conventional evaluation of EEG data considers s wavelength of at least 50 ms, experts are content with this type of data. The standard preprocessing is a low-pass filtering with a cutoff of 40 Hz, say. For determining the persistence or turning rate at d≤8ms, or frequency ≥130Hz, however, the 50-Hz noise of the power net is a real obstacle. This also holds for the ECG.

**Figure 21:**
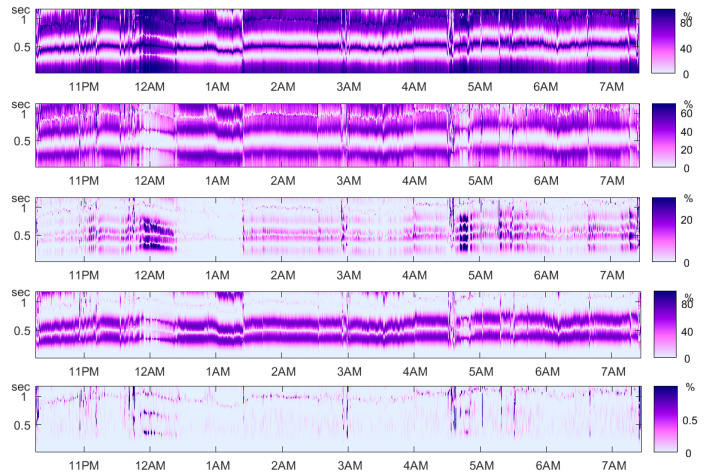
Theorem 1 for the plethysmogram over a whole night. From top to bottom: $\tilde{\tau },\tilde{\beta },\tilde{\gamma },\tilde{\delta }.$ The bottom panel contains the error of the equation of Theorem 1 on a scale from 0 up to 1%. Data of Person n3 from Terzano et al. [23].

The plethysmogram is determined by a light sensor and can be measured without power noise contamination. Nevertheless, it is mostly measured at low frequencies like 30 Hz, because of its smooth appearance. Perhaps there is also a fine structure for higher resolution that can be studied by order methods. Figure 21 shows the validity of Theorem 1 for a plethysmogram of [23].

## 10. Conclusions

Today’s time series of weather, geophysical and environmental observations, and from medical monitoring, are huge, covering several time scales of interest. Sliding windows analysis is an appropriate method, known from the spectrogram of speech data. Here we considered four order correlation functions instead of the spectrum. Their calculation is fast and simple, based on weak stationarity assumptions, and robust with respect to outliers and missing values. We studied the properties of the order correlation functions and demonstrated how they describe new aspects of the structure of the data.

## Figures and Tables

**Table 1:** Range and extreme cases for various correlation functions.

Function	Range	Min Assumed for	Max Assumed for
Autocorrelation ρ	[−1,1]	linear decreasing series	linear increasing series
Spearman rank autocorr.	[−1,1]	decreasing series	increasing series
Up-down balance β	[−1,1]	decreasing series	increasing series
Persistence τ	$\left[-\frac{1}{3},\frac{2}{3}\right]$	alternating series	monotone series
Turning rate TR	[0,1]	monotone series	alternating series
Up-down scaling δ	[−1,1]	$-(t+{\left(-1\right)}^{t})$	$t+{\left(-1\right)}^{t}$
Rotational asymmetry γ	[−1,1]	${\left(-2\right)}^{t}$	${\left(-\frac{1}{2}\right)}^{t}$

**Table 2:** Invariance of correlation functions under symmetries.

Function	Time Reversal	Negative Function	Rotation
ρ, Spearman, τ	+	−	−
β and δ	−	−	+
Rotational asymmetry γ	−	+	−

**Table 3:** Behavior of correlation functions of periodic series.

Function	Half Period $\frac{L}{2}$	Period *L*	Symmetry Type
ρ, Spearman,	minimum	maximum	vertical line
Persistence τ	minimum	bumped maximum	vertical line
β,γ, and δ	zero	zero or discontinuity	symmetry center

## References

[1] Amigo J., Keller K., Kurths J. (2013). Recent progress in symbolic dynamics and permutation complexity. Ten years of permutation entropy. Eur. Phys. J. Spec. Top..

[2] Amigo J.M. (2010). Permutation Complexity in Dynamical Systems.

[3] Zanin M., Zunino L., Rosso O., Papo D. (2012). Permutation entropy and its main biomedical and econophysics applications: A review. Entropy.

[4] Carpi L.C., Saco P.M., Rosso O.A. (2010). Missing ordinal patterns in correlated noises. Phys. A.

[5] Martinez J.H., Herrera-Diestra J.L., Chavez M. (2018). Detection of time reversibility in time series by ordinal patterns analysis. Chaos.

[6] Zanin M., Rodríguez-González A., Menasalvas Ruiz E., Papo D. (2018). Assessing time series reversibility through permutation patterns. Entropy.

[7] Parlitz U., Berg S., Luther S., Schirdewan A., Kurths J., Wessel N. (2012). Classifying cardiac biosignals using ordinal pattern statistics and symbolic dynamics. Comput. Biol. Med..

[8] McCullough M., Small M., Iu H., Stemler T. (2017). Multiscale ordinal network analysis of human cardiac dynamics. Philos. Trans. R. Soc. A Math. Phys. Eng. Sci..

[9] Bandt C., Shiha F. (2007). Order patterns in time series. J. Time Ser. Anal..

[10] Bandt C., Rojas I., Pomares H. (2015). Permutation entropy and order patterns in long time series. Time Series Analysis and Forecasting.

[11] Bandt C. (2017). A new kind of permutation entropy used to classify sleep stages from invisible EEG microstructure. Entropy.

[12] Bandt C., Pompe B. (2001). Permutation entropy: A natural complexity measure for time series. Phys. Rev. Lett..

[13] Bandt C. (2014). Autocorrelation type functions for big and dirty data series. arXiv.

[14] Rosso O., Larrondo H., Martin M.T., Plastino A., Fuentes M. (2007). Distinguishing Noise from Chaos. Phys. Rev. Lett..

[15] López-Ruiz R., Nagy Á., Romera E., Sañudo J. (2009). A generalized statistical complexity measure: Applications to quantum systems. J. Math. Phys..

[16] Deutscher Wetterdienst. Climate Data Center. ftp://ftp-cdc.dwd.de/pub/CDC/observations_germany.

[17] Brockwell P., Davies R. (1991). Time Series, Theory and Methods.

[18] Shumway R., Stoffer D. (2006). Time Series Analysis and Its Applications.

[19] Bandt C. (2017). Crude EEG parameter provides sleep medicine with well-defined continuous hypnograms. arXiv.

[20] Ferguson S., Genest C., Hallin M. (2000). Kendall’s tau for serial dependence. Can. J. Stat..

[21] National Oceanic and Atmospheric Administration National Water Level Observation Network. https://www.tidesandcurrents.noaa.gov/nwlon.html.

[22] California Air Resources Board. www.arb.ca.gov/adam.

[23] Terzano M., Parrino L., Sherieri A., Chervin R., Chokroverty S., Guilleminault C., Hirshkowitz M., Mahowald M., Moldofsky H., Rosa A. (2001). Atlas, rules, and recording techniques for the scoring of cyclic alternating pattern (CAP) in human sleep. Sleep Med..

[24] Goldberger A., Amaral L., Glass L., Hausdorff J., Ivanov P., Mark R., Mietus J., Moody G., Peng C.K., Stanley H. (2000). PhysioBank, PhysioToolkit, and PhysioNet: Components of a new research resource for complex physiologic signals. Circulation.

